# Public Protests and the Risk of Novel Coronavirus Disease Hospitalizations: A County-Level Analysis from California

**DOI:** 10.3390/ijerph18189481

**Published:** 2021-09-08

**Authors:** Linh N. Bui, Rachel L. Berkowitz, Wendy Jilek, Andrew J. Bordner, Kristen M. J. Azar, Alice Pressman, Robert J. Romanelli

**Affiliations:** 1 Department of Nursing, School of Natural Sciences, Mathematics, and Engineering, California State University, Bakersfield, CA 93311, USA; 2 Institute for Advancing Health Equity, Sutter Health, Palo Alto, CA 94301, USA; AzarK@sutterhealth.org (K.M.J.A.); PressmAR@sutterhealth.org (A.P.); Rob_Romanelli@randeurope.org (R.J.R.); 3 Department of Public Health and Recreation, College of Health and Human Sciences, San José State University, San Jose, CA 95192, USA; 4 Health Services Advisory Group, Phoenix, AZ 85016, USA; wmjilek@berkeley.edu; 5 Center for Health Systems Research, Sutter Health, Walnut Creek, CA 94596, USA; 6 Design & Innovation, Sutter Health, San Carlos, CA 94070, USA; bordnea@sutterhealth.org; 7 Department of Epidemiology and Biostatistics, University of California, San Francisco, CA 94158, USA

**Keywords:** novel coronavirus disease (COVID-19) hospitalization, public protests, California, mixed-effects models, county-level

## Abstract

The objective of this study was to assess the relationship between public protests and county-level, novel coronavirus disease (COVID-19) hospitalization rates across California. Publicly available data were included in the analysis from 55 of 58 California state counties (29 March–14 October 2020). Mixed-effects negative binomial regression models were used to examine the relationship between daily county-level COVID-19 hospitalizations and two main exposure variables: any vs. no protests and 1 or >1 protest vs. no protests on a given county-day. COVID-19 hospitalizations were used as a proxy for viral transmission since such rates are less sensitive to temporal changes in testing access/availability. Models included covariates for daily county mobility, county-level characteristics, and time trends. Models also included a county-population offset and a two-week lag for the association between exposure and outcome. No significant associations were observed between protest exposures and COVID-19 hospitalization rates among the 55 counties. We did not find evidence to suggest that public protests were associated with COVID-19 hospitalization within California counties. These findings support the notion that protesting during a pandemic may be safe, ostensibly, so long as evidence-based precautionary measures are taken.

## 1. Introduction

The novel coronavirus disease (COVID-19) pandemic, caused by the Severe Acute Respiratory Syndrome Coronavirus 2 (SARS-CoV-2), has ravaged communities across the United States (U.S.). Between 23 January 2020 and 26 August 2021, the U.S. recorded more than 38 million cumulative cases and 630,000 related deaths [1].Since the virus can be transmitted by pre-symptomatic and asymptomatic carriers [2], strategies to mitigate the spread of the virus will continue to be central to the public health response alongside vaccine roll-out. Such mitigation strategies to reduce SARS-CoV-2 transmission have included stay-at-home orders, mask-wearing, and social-distancing guidelines, as well as contact tracing and self-quarantining [3,4,5,6]. Within the U.S. and around the world, these strategies and the realities of the COVID-19 pandemic have both affected and highlighted existing inequities in the environments of cities and towns, calling into question how such places should evolve as a result of the pandemic (referred to in some contexts as the “post-COVID city”) [7,8,9]. Within this wider conversation, a tension remains between the recognized need for ongoing access to public space to support physical and mental wellbeing and the importance of practicing and enforcing stay-at-home orders, social distancing, and masking to protect individuals from airborne disease spread [10,11].

Simultaneously, against the backdrop of an especially contentious political climate in the U.S., the country also witnessed a dramatic increase in participation in public protests. Globally, the stress of the pandemic and related preventive measures intersected with long-standing grievances and cries of injustice, resulting in a worldwide increase in protests in 2020 [12,13,14]. In the U.S., March–September 2020 saw nearly twice the number of protests nationwide as compared with the same time period in 2019 [15]. Over 800 protests against pandemic interventions (e.g., mask-wearing, business closures, school closures) took place across the U.S. [15], including the wave of “Liberate” protests in March and April, encouraged in part by tweets from then President Donald Trump [16]. The killing of George Floyd by Minneapolis police on 25 May 2020 fueled already prevalent outrage over police violence against Black Americans [17,18] and led to protests for racial justice that surged alongside protests in response to economic and political tensions of the pandemic. Between March and September 2020, racial justice accounted for 65% of protests reported in the database, as compared to only 6% in 2019 [15].

Protests often result in the gathering of large groups of individuals shouting and crowding together for an extended period of time, activities viewed as counterproductive to preventing the transmission of SARS-CoV-2 [18,19,20,21,22]. Despite these concerns, there is overwhelming recognition of the importance of protecting first amendment rights to peaceful protest. In early summer of 2020, public health officials penned an open letter with over 1200 signatures advocating for risk mitigation strategies to support the health of protesters [20]. The authors encouraged protesters at gatherings to wear masks and, to the extent possible, social distance, and to stay home if sick. The authors further insisted that law enforcement at protests refrain from using crowd dispersion techniques, such as tear gas and pepper spray, which may increase coughing, among other harmful outcomes, thereby increasing the risk of viral transmission [18,23]. However, it is recognized that individuals protesting against pandemic intervention may be less likely to observe recommended practices to prevent transmission of the virus [22].

Understanding whether public protests impact community spread of SARS-CoV-2 can help to inform strategies and policies to ensure the safety of protesters and their communities both now and in the future. Such research is in its infancy. To date, we have identified three such studies, all of which focused exclusively on anti-racist protests in the wake of the killing of George Floyd [19,24,25]. First, a working paper from the National Bureau of Economic Research (NBER) examining 208 U.S. counties encompassing 315 large cities did not find a significant relationship between Black Lives Matter protests and COVID-19 daily case growth rates 21 or more days following the beginning of protests [19]. Second, an event-study analysis of eight U.S. cities in states with rescinded or expired stay-at-home orders and protests in the tens of thousands found statistically significant higher-than-expected SARS-CoV-2 infection growth rates in six cities [24]. Third, a bivariate analysis comparing case rates in counties with Black Lives Matter protests with control counties found that one, two, and three weeks after a protest there was a small but significant increase in COVID-19 case rates, though the authors suggest other factors likely explain this otherwise minimal finding [25].

There are limitations to these prior analyses, as case numbers and growth rates may be impacted by changes in testing availability and accessibility over time [26]. COVID-19 hospitalizations, however, are less sensitive to testing challenges and are more likely to be reported and thus, represent a more stable outcome measure [27]. To date, no published articles have examined the relationship between protests and COVID-19 hospitalizations. The two studies that found associations between increases in COVID-19 cases and protests did not account for other potential confounding factors (e.g., mobility, county characteristics) in their analyses [24,25]. Moreover, all three studies focused on time periods early in the pandemic and did not assess all types of protests taking place over time. The conflicting results and limitations of the existing studies underscore the importance of additional, robust analyses to further examine whether protests have any relationship with the community spread of COVID-19. 

This study investigates the relationship between the protests occurring in California state counties and county-level COVID-19 hospitalization rates. As the nation’s most populous state, California has been profoundly impacted by the pandemic. As of 26 January 2021, California had the most COVID-19 cases in the U.S. (over 3 million cases, 12% of reported cases nationwide) and the largest number of COVID-19-related deaths (37,188 deaths, 9% of deaths nationwide) [1]. California is also considered a politically active and progressive state. According to the aforementioned protest database, California accounts for the largest absolute number of protests since the site began collecting data in 2017, representing 11% of all recorded demonstrations in the country [15]. California’s counties vary widely in size and density: California’s 58 counties range in size from approximately 1000 residents to over 10 million with the number of housing units ranging from an estimated 1767 to over 3 million [28]. 

Given the potential increased risk of SARS-CoV-2 transmission during protest activity, we hypothesized that the occurrence of any protests on a given day within a CA county as well as the number of protests on a given day is associated with an increased COVID-19 hospitalization rate two weeks later. The results from this study can inform actions and policies to protect protesters, and surrounding communities, in California and across the country, during the current and future pandemics. 

## 2. Materials and Methods

Three California counties were excluded from the analysis: two due to lack of available COVID-19 hospitalization data (Alpine and Sierra Counties) and one due to no hospitalizations reported (Sutter County) during the study period. The final sample included 55 of 58 California counties with 11,000 county-days during the study period, 29 March–14 October 2020.

### 2.1. Outcome and Exposure Measurements

COVID-19 hospitalization was measured by the daily number of hospitalized COVID-19 patients (including suspected and confirmed hospitalized COVID-19 cases) in each county, using data from the California Department of Public Health [29]. 

Protests, as the main exposure, was obtained from Count Love (downloaded on 15 October 2020) [15], an online database that uses local newspaper and television outlets to document when and where protest occurred in the U.S, the number of attendees, and common protested issues [30]. Two protest variables were operationalized: a binary variable of any protest (no protest vs. any protest) and a categorical variable representing the number of protests on a given day in a county (no protests vs. 1 or >1 protest). 

For descriptive purposes, protests were further categorized by protest type as “for racial/social justice”, “counter racial/social justice”, “for pandemic interventions”, “against pandemic interventions”, and “miscellaneous” based on corresponding tagged phrases and confirmatory examination of the affiliated articles in the Count Love database (see Supplementary Table S1 for further description of categorizations). 

### 2.2. Covariates

Covariates, including daily county mobility, county-level characteristics, and time trends, were identified a priori as potential confounders for the relationship between protests and COVID-19 hospitalization rates across counties. 

### 2.3. County Mobility

Since social distancing is a known mitigation strategy to reduce COVID-19, we used county mobility as a proxy for county population social distancing practices. Daily mobility data were obtained from SafeGraph [31], a company that uses GPS pings from anonymous mobile devices to estimate different social distancing metrics at census block group level. Using suggested methods from SafeGraph, we created a continuous county-level mobility index which is a weighted fraction of the devices inferred to have remained at home throughout the entire day in a given county [32].

### 2.4. County-Level Characteristics

County demographic characteristics were obtained from the American Community Survey (ACS) 2014–2018 [33], and factors related to increased risk for SARS-CoV-2 exposure and COVID-19 morbidity and mortality were identified [34,35]. These factors included percentages of the population that are male, Non-Hispanic Black, Non-Hispanic American Indian/Alaska Native, or Hispanic, as well as the median age of the county. 

Percentages of individuals with comorbidities known to increase risk of COVID-19 morbidity and mortality were also obtained [36]: age-adjusted percentage of adults (20+) who had diabetes (2017) from the United States Diabetes Surveillance System [37], percentage of adults (18+) of who are both current smokers and have smoked at least 100 cigarettes in their lifetime (2017) from the Behavioral Risk Factor Surveillance System [38], and percentage of adults (20+) who were obese (2016) from the United States Diabetes Surveillance System [38]. The continuous variable of percent of urban housing units from the 2010 Decennial Census [39] accounted for potential variation in crowding across counties. 

Potential variation in neighborhood environments was obtained through the Healthy Places Index (HPI) [40] developed by the Public Health Alliance of Southern California. The HPI is a continuous weighted score that combines the economic, education, housing, healthcare access, environment, neighborhood, social and transportation characteristics of a census tract to predict life-expectancy at birth, with the highest score identifying the healthiest tract environment. The Alliance provided county-level HPI estimates based on a population-weighted average of included census tracts. 

Finally, given the politicized nature of both the pandemic and protests, U.S. political affiliation is likely to affect where and if protests occur, behaviors to mitigate viral transmission, and the outcome of COVID-19 hospitalizations. To account for this, information on county-level voting data was obtained from the MIT Election Data and Science Lab [41] and a binary variable was creating indicating political persuasion. If a larger proportion of the county’s votes in the 2016 presidential election were for Hilary Rodham Clinton, the county was considered to lean Democratic. 

### 2.5. Time Trends

Based on an examination of the time trend of COVID-19 hospitalization across counties during the study period, daily time trends were divided into three time periods: (1) before 1 June, (2) 1 June–21 July, and (3) after 22 July. Three spline variables were created to represent the three time periods in our models based on visual observation of average changes in county hospitalization rates over time (see Supplementary Figure S1 for observed vs. predicted COVID-19 hospitalization rates using this time trend). The knots (1 June and 21 July) were also selected since they provided the best model fit to predict COVID-19 hospitalization using the Bayesian information criterion (BIC). 

### 2.6. Descriptive Analyses

To explore patterns in the data, graphs were used to describe (1) the daily average COVID-19 hospitalization rate and the total number of protests across the total sample of 55 counties, and (2) the proportion of protests by protest type, overall and for the counties with any protests. Descriptive analyses of COVID-19 hospitalization rates, protests, and mobility data across the county-days and county-level covariates across 55 counties, summarized using mean and standard deviation or number and frequency, were conducted. 

### 2.7. Statistical Modelling

Mixed-effect negative binomial regression models were used to examine the association between each protest exposure and COVID-19 hospitalization rates two weeks later. Based on estimates of a median incubation period of 4 days [42] and estimates of the time between symptom onset and hospitalization in one study ranging from a median of 3 to 10.4 days [43] and a mean of 2 to 6.5 days in another study [44], assessed hospitalization rates at one week, two weeks, three weeks, and four weeks post potential protest exposure. We present the two-week post-protest lag as our primary analysis, with one week, three week, and four week lags analyzed as sensitivity analyses. Models included random-effects for each county and fixed-effect covariates as described above. We controlled for the mobility index 15 days prior to the hospitalization to characterize the social distancing practice in a county prior to protests. Collinearity assessment revealed that HPI and comorbidity variables (diabetes, obesity, and smoking) were all highly correlated but did not alter the coefficients or significance levels meaningfully, and so all were included to account for a priori defined confounders. County population from the ACS 2014–1028, was included as an offset variable to model COVID-19 hospitalization rates [33].

Robust standard errors were used to account for heteroscedasticity of the errors. Coefficients were reported as incidence rate ratios (IRR) with 95% confidence intervals (CI); a *p*-value < 0.05 was considered statistically significant. Data analysis was conducted using Stata 16 [45] and R [46]. Since all data were county-level and de-identified, this study was not classified as human subjects research. Thus, institutional review board approval was not required.

### 2.8. Sensitivity Analyses

To account for potential shorter or longer incubation periods for SARS-CoV-2 infection, sensitivity analyses were conducted with a one-week lag, three-week lag, and four-week lag of COVID-19 hospitalization, with mobility data for 8 days, 22 days, or 29 days prior to hospitalization, respectively, included as confounders. In addition, to account for the possibility that the type of protest might confound the relationship between county-level protest days and county-level hospitalization rates, we conducted two additional analyses. The first used a categorical variable measuring whether a county-day included any “against pandemic” protest, no “against pandemic” protests, or no protests (reference) as the main exposure variable in the fully adjusted model across all county-days. The second included in a categorical variable of each protest type (“for racial/social justice”, “counter racial/social justice”, “for pandemic interventions”, “against pandemic interventions”, or “miscellaneous” (see Supplementary Table S1 for description of each category)) in the fully adjusted model for the exposure variable of any versus no protests across a subset of county-days with either one protest or no protests. Finally, to check whether our results were influenced by outliers, the relationship between protests and COVID-19 hospitalization was estimated after the removal of three small non-protest counties—Inyo, population 18,085; Modoc, population 8938; and Mono, population 14,174 [33], all which have days with disproportionately high COVID-19 hospitalization rates (>100 hospitalizations per 100,000 individuals) at the very beginning of the time period (29 March–mid-April). 

## 3. Results

### 3.1. Descriptive Findings

**Figure 1:**
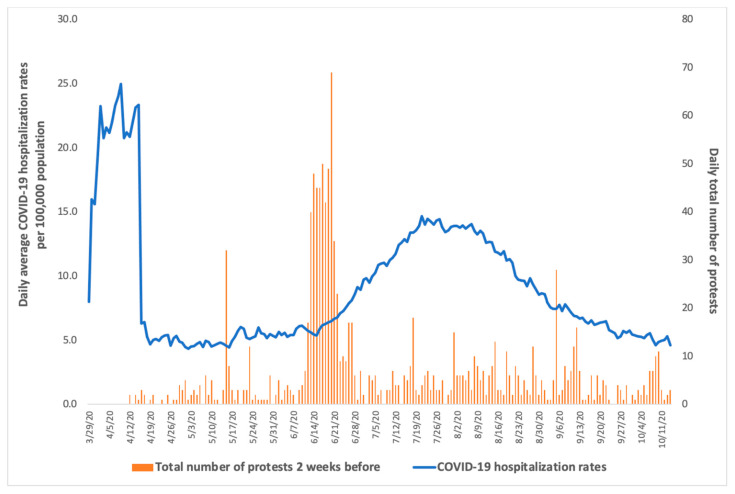
Daily average COVID-19 hospitalization rate per 100,000 population and total number of protests two weeks earlier from 29 March–14 October 2020 across California state counties (*n* = 55 counties).

Across the 55 counties, average COVID-19 hospitalization rates peaked within the range of 20–25 cases per 100,000 population during the first two weeks of the study period, and then gradually decreased (Figure 1). Hospitalization rates sharply increased again starting from 1 June, peaked by the end of the third week of July, and then decreased until 14 October 2020. Daily total number of protests in all counties were less than 10 protests per day before the end of May (with the exception of one day in late April when over 30 protests occurred), at which point the total number of protests in all counties sharply increased during the first two weeks of June, with multiple days when 40–50 protests occurred and one day that saw nearly 70 protests across the state, before returning to near pre-late-May daily totals. 

Average daily COVID-19 hospitalization rate per county was 9.3 per 100,000 population (SD = 29.7) (Table 1). On average, there were 14 days when protests occurred, with 1–2 protests occurring per day and an average total number of protests during the study period per county of 23. Average mobility index (average fraction of devices staying at home throughout the entire day) per county-day was 34.4% (SD = 6.0%). An average of 8.8% of adults had diabetes, 27.4% of adults were obese, and 12.1% of adults were current smokers. Population size ranged widely across the 55 counties with an average of 709,978 persons (SD = 1,501,872), the counties were on average mostly urban (Mean = 71.6%, SD = 27.0%), and the majority of counties leaned Democrat (*n* = 32, 58.2%). 

**Figure 2:**
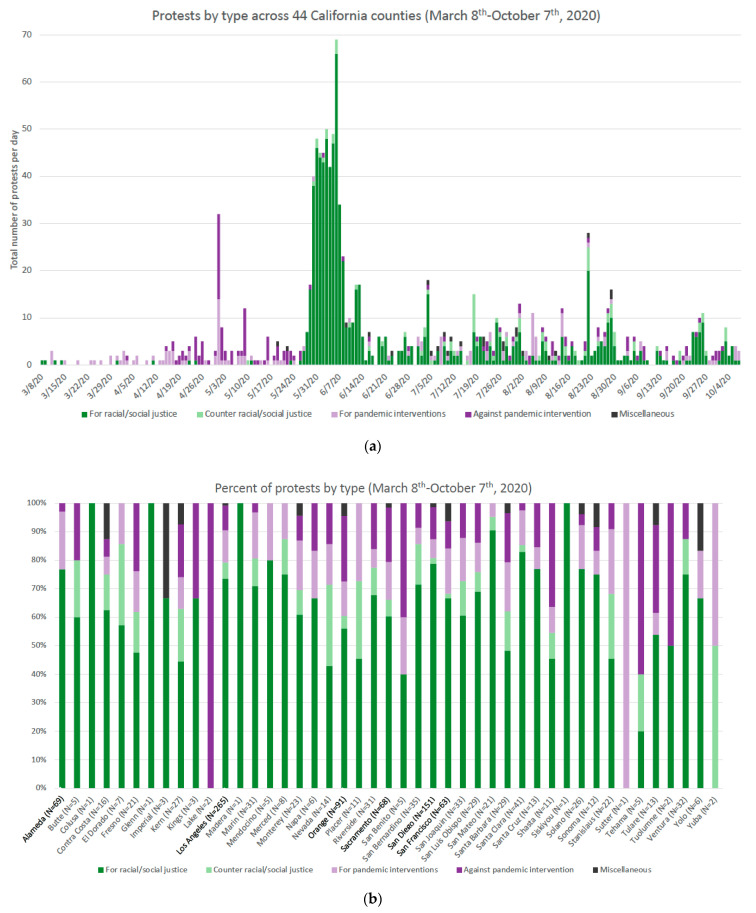
Protest by type over time (**a**) and by county (**b**). N: Total number of protests for a given county during the time period, with the names of counties with >50 protests bolded.

Protests occurred in 44 of the 55 California counties in the study (80%). The majority of protests prior to the end of May focused on the pandemic, including protests both for and against pandemic intervention (Figure 2). Beginning the end of May and coinciding with the surge of protests, the majority of protests were for racial/social justice. While protest numbers returned to lower levels in mid-June, protests for racial/social justice remained prevalent at higher rates than during the pre-May period. Most counties include a mixture of protest types during the time period. In counties with the largest numbers of protests (Los Angeles, *n* = 265 protests; San Diego, *n* = 151; Orange, *n* = 91; Alameda, *n* = 69; Sacramento, *n* = 68; and San Francisco, *n* = 63), protests for racial/social justice constituted the majority.

### 3.2. Findings from Statistical Models

In a given county, among a total of 55, there was no statistically significant association between COVID-19 hospitalization rates and any protests (IRR: 0.953, 95% CI: 0.899, 1.020; Model 1). There was no statistically significant association between COVID-19 hospitalization and 1 protest (IRR: 0.953, 95% CI: 0.891, 1.019; Model 2) or >1 protest (IRR: 0.951, 95% CI: 0.867, 1.044; Model 2) (reference = no protests) in fully adjusted models (Table 2). 

### 3.3. Findings from Sensitivity Analyses

There was no significant association between either exposure variable and COVID-19 hospitalization rates one week, three-weeks, or four weeks following protests (Supplementary Table S2). Additionally, no statistically significant relationship was identified between protests and COVID-19 hospitalization rates two-weeks in either sensitivity analysis including protest type (data not shown). Finally, in sensitivity analyses among 52 counties without outlier rates of COVID-19 hospitalizations, there was a statistically significant association between COVID-19 hospitalization rates and any protest (IRR: 0.925, 95% CI: 0.898, 0.975) and 1 protest (IRR: 0.926, 95% CI: 0.879, 0.975) vs. no protest, but not for >1 protests vs. no protests (IRR: 0.923, 95% CI: 0.851, 1.001) (Supplementary Table S3). 

## 4. Discussion

Counter to our hypothesis, between 29th March and 14th October 2020, any protest or number of protests were not associated with increased rate in COVID-19 hospitalizations across 55 California counties (regardless of a lag of one, two or three weeks), in fact, trends were in the opposite direction (protests were associated with fewer hospitalizations). Point estimates were of similar magnitude, but were statistically significant, after removing three counties with outlier hospitalization rates, suggesting, on average, an approximate 7% decreased COVID-19 hospitalization rate two-weeks after any protests or one protest as compared with no protests, independent of all covariates included in the model.

Our study is one of only a few that have examined the potential impact of protests on COVID-19 outcomes and contributes uniquely to this growing body of literature. Our main finding of no significant relationship between protests and COVID-19 hospitalization rates is aligned with the findings in the NBER study by Dave et al. [19], but is counter to the significant increases in COVID-19 cases in studies by Valentine et al. [24] and Neyman et al. [25]. That our findings were statistically significant after removing outlier counties likely reflects the increased precision of our estimates, rather than a meaningfully reduced risk (or protective effect) of public protests for COVID-19 hospitalization. Unlike previous studies that focused on COVID-19 cases [19,24,25], our study focused on COVID-19 hospitalization rates—an outcome that is not sensitive to local testing access and availability over time or across counties, and speaks to severe COVID-19 cases. Our study also includes an extended time period during which to evaluate the potential relationship between protests and COVID-19 outcomes (200 days, between 29 March–14 October 2020). Finally, we chose to consider all protests since the protest activities that are hypothesized to increase risk of exposure and transmission may occur across all types of protests [19,20,21,22]. This is in contrast to previous studies that focused exclusively on Black Live Matters protests [19,24,25].

There are several possible explanations for the seemingly protective relationship between protests and COVID-19 hospitalization rates we observed. One explanation relates to increased protective measures taken by protestors themselves. News reports and the promotion of COVID-19 risk/harm reduction strategies by entities such as the Movement for Black Lives [47] all suggest that participants in racial/social justice protests (the majority of protests in our study sample, see Figure 2) may have been likely to implement evidence-based practices to limit the risk of SARS-CoV-2 exposure and transmission [20]. Additional vigilance in anticipation of and in response to protesting with respect to testing, mask-wearing, and quarantining may therefore represent unmeasured confounders and/or mediators of the relationship between protests and COVID-19 hospitalization rates. Research focused on the experiences and decisions of protestors is needed to understand these potential pathways. In addition, such intentional harm-reduction strategies may be less likely to occur during protests against pandemic interventions or in favor of rhetoric that downplay the seriousness of COVID-19 [22]. Additional research to effectively distinguish between the influences of different protest types on behaviors that may increase transmission of an airborne infectious agent is necessary to unpack the potential differential risk. 

Another possibility is that non-protest-participating residents may have altered their own behaviors and reduced their mobility in response to the protests, to avoid interaction with protestors. Dave et al. found a significant relationship between protests and reduced mobility four to seven days after protests occurred, though their study did not assess whether mobility mediated the relationship between protests and COVID-19 cases [19]. A full mediation analysis was beyond the scope of our study. Further investigation is necessary to understand whether reduced mobility in response to protests may help explain the association of protests with reduced COVID-19 hospitalization rates.

There are several important limitations of our study. Protest data from Count Love is based on what is reported in mined news articles. It is possible that protests that did not receive media attention may be meaningfully different from those that were reported. We considered examining protest type as an exposure variable but recognized that it would be difficult to discern the impacts of different types of protests occurring on a given day in a county. We also considered protest size as a potential exposure, but ultimately decided that the data quality was inadequate. Though we were intentional and thoughtful regarding the a priori specified confounders we included in our analysis, it is possible that there are unmeasured confounders that may explain aspects of our results. It is possible that there was a source of autocorrelation at play; however, neglecting to adjust for this lag would have led to a larger observed effect (bias away from the null). Since we found no significant effect, there was no added benefit to including such a term. Finally, our results are specific to California and may not be generalizable to other states; however, our analytic approach is replicable and can inform analyses for other states.

## 5. Conclusions

During this and future pandemics, the ability to use protests as one methodology to advocate for change remains vital. Our study did not find evidence of increased COVID-19 hospitalization rates after protests occurred in California counties. While the limitations noted underscore the need for caution with interpreting a causal relationship from our findings, the results do support the notion that it is possible to protest safely in the midst of a pandemic, ostensibly, so long as evidence-based precautionary measures are taken [48]. As the pandemic continues into its second year, public demonstrations related to COVID-19 and societal issues remain fixtures of daily life around the world [49]. Further research is needed to understand both the individual-level relationship between protest participation, SARS-CoV-2 exposure, and COVID-19 hospitalization, morbidity, and mortality, particularly with more virulent strains such as the Delta variant resulting in breakthrough cases among vaccinated individuals [50]. In addition, research focused on the impacts of interventions to ensure preventive measures against SARS-CoV-2 transmission are taken during protests is needed to inform the policies and practices health departments should implement to ensure that the right to protest remains protected and safe. Alongside calls for more equitable and public-health-centered development in a post-COVID-19 context [7,8,9], our study highlights the need for research and practice to consider ways in which public protest can continue safely.

## Figures and Tables

**Table 1:** COVID-19 hospitalization rate, protest data, and covariates for all counties and county-days (29 September–14 October 2020).

	Mean (SD) ^1^
Daily COVID-19 hospitalization per 100,000 population per county	9.3 (29.7)
**County-day characteristics (*n* = 11,000 county days)**	
**Protests**	
Counties with any protests (*n* (%))	44 (80.0)
Avg. number of days with any protests per county	14.3 (19.2)
Total number of protests during study period per county	22.9 (42.8)
Daily number of protests (among protest days) per county	1.6 (1.5)
**Daily Mobility Index**	
Average proportion of devices staying at home throughout the entire day	34.4 (6.0)
**County characteristics (*n* = 55 counties)**	
Healthy Place Index ^2^	−0.039 (0.308)
**Comorbidities**	
Percentage of adults with diabetes	8.8 (2.6)
Percentage of adults with obesity	27.4 (5.8)
Percentage of adults smoking ^4^	12.1 (1.8)
**Demographic characteristics**	
Total population	709,978 (1,501,872)
Percentage of male population	50.5 (2.5)
Median age	39.3 (6.2)
**Percentage of race/ethnic groups**	
Hispanic	31.1 (18.1)
Black ^3^	3.1 (3.0)
AI/AN ^4^	1.3 (1.9)
**Percentage of urban housing units**	71.6 (27.0)
**Political leaning**	
Democrat (*n* (%))	32 (58.2)

Bold text headings in body of table indicate a group of characteristics or variables. ^1^ SD = Standard deviation ^2^ Higher value = “Healthier” place (structurally); ^3^ Non-Hispanic Black or African American; ^4^ Non-Hispanic American Indian/Alaska Native; 12 county days across 7 counties missing hospitalization values.

**Table 2:** Incidence rate ratios (IRR) and 95% Confidence Interval (95% CI) from multivariable mixed-effect negative binomial models: Association between protests and two-week post-protest COVID-19 hospitalization across California counties (29 March–14 October 2020).

	All Counties (*n* = 55 Counties)	
	Model 1	Model 2
	IRR (95% CI)	IRR (95% CI)
Any protests	0.953 (0.889; 1.020)	--
No protests	1.00	--
1 protest	--	0.953 (0.891; 1.019)
>1 protest	--	0.951 (0.867; 1.044)
No protests	--	1.00
% of devices staying at home	0.974 * (0.953; 0.994)	0.974 * (0.953; 0.994)
Healthy Places Index	0.794 (0.259; 2.439)	0.794 (0.258; 2.439)
% with diabetes	1.041 (0.937; 1.156)	1.041 (0.937; 1.156)
% obese	0.956 * (0.921; 0.993)	0.956 * (0.921; 0.993)
% smokers	0.892 (0.741; 1.074)	0.892 (0.741; 1.074)
% male	0.950 (0.892; 1.013)	0.950 (0.892; 1.013)
Median age	1.028 (0.944; 1.120)	1.028 (0.944; 1.120)
% Hispanic	1.035 * (1.018; 1.053)	1.035 * (1.018; 1.053)
% Black ^1^	1.093 * (1.049; 1.139)	1.093 * (1.049; 1.139)
% AI/AN ^2^	1.226 * (1.095; 1.372)	1.226 * (1.095; 1.372)
% of urban housing units	1.021 * (1.005; 1.037)	1.021 * (1.005; 1.037)
Democratic county	0.624 * (0.406; 0.959)	0.624 * (0.406; 0.960)
Republican county	1.00	1.00
Spline 1 (before 1 June)	0.987 * (0.978; 0.996)	0.987 * (0.978; 0.996)
Spline 2 (1 June–21 July)	1.040 * (1.026; 1.053)	1.040 * (1.026; 1.053)
Spline 3 (22 July–14 October)	0.960 * (0.954; 0.967)	0.960 * (0.954; 0.967)

^1^ Non-Hispanic Black or African American; ^2^ Non-Hispanic American Indian/Alaska Native; * *p* < 0.05.

## Data Availability

COVID-19 hospitalization data comes from publicly available data from the California Department of Public Health. Protest data was obtained from the publicly available website Count Love. Publicly available daily mobility data were obtained from SafeGraph. County demographic characteristics were obtained from the publicly available American Community Survey (ACS) 2014–2018. Age-adjusted percentage of adults (20+) who had diabetes (2017) was obtained from the publicly available United States Diabetes Surveillance System. Percentage of adults (18+) of who are both current smokers and have smoked at least 100 cigarettes in their lifetime (2017) and percentage of adults (20+) who were obese (2016) were obtained from the publicly available County Health Rankings and Roadmaps. The Healthy Places Index was obtained from the publicly available Healthy Places Index website, with support from the Public Alliance of Southern California to develop county-level estimates. County-level voting data was obtained from MIT Election Data and Science Lab.

## Supplementary Materials

The following are available online at https://www.mdpi.com/article/10.3390/ijerph18189481/s1. Table S1: Description of Count Love tag series included in author-generated protest-type categories; Figure S1: Observed versus predicted COVID-19 hospitalization rates using time-trend spline, averaged across all 55 counties and for each county, using mixed negative binomial models of the association between having any protests and two-week post-protest COVID-19 hospitalization; Table S2: Incidence rate ratios (IRR) and 95% Confidence Interval (95% CI) from multivariable mixed negative binomial models: Association between protests and one-week and three-week post-protest COVID-19 hospitalization across 55 California counties, 29 March–14 October 2020; and Table S3: Incidence rate ratios (IRR) and 95% Confidence Interval (95% CI) from multivariable mixed negative binomial models: Association between protests and two-week post-protest COVID-19 hospitalization across 51 California counties (after removing 3 counties with outliers of hospitalization), 29 March–14 October 2020.
