# Supplementary material for: Public Protests and the Risk of Novel Coronavirus Disease Hospitalizations: A County-Level Analysis from California
Source: Int J Environ Res Public Health. 2021 Sep 8;18(18):9481. doi: 10.3390/ijerph18189481 (PMC8467497; doi:10.3390/ijerph18189481)
Supplement: Supplementary file 1 [file ijerph-18-09481-s001.zip › Supplementary Figure S1.pdf]

Supplementary Figure S1: Observed versus predicted COVID-19 hospitalization rates using time-trend spline, averaged across all 55 counties and for each county, using mixed negative binomial models of the association between having any protests and two-week post-protest COVID-19 hospitalization

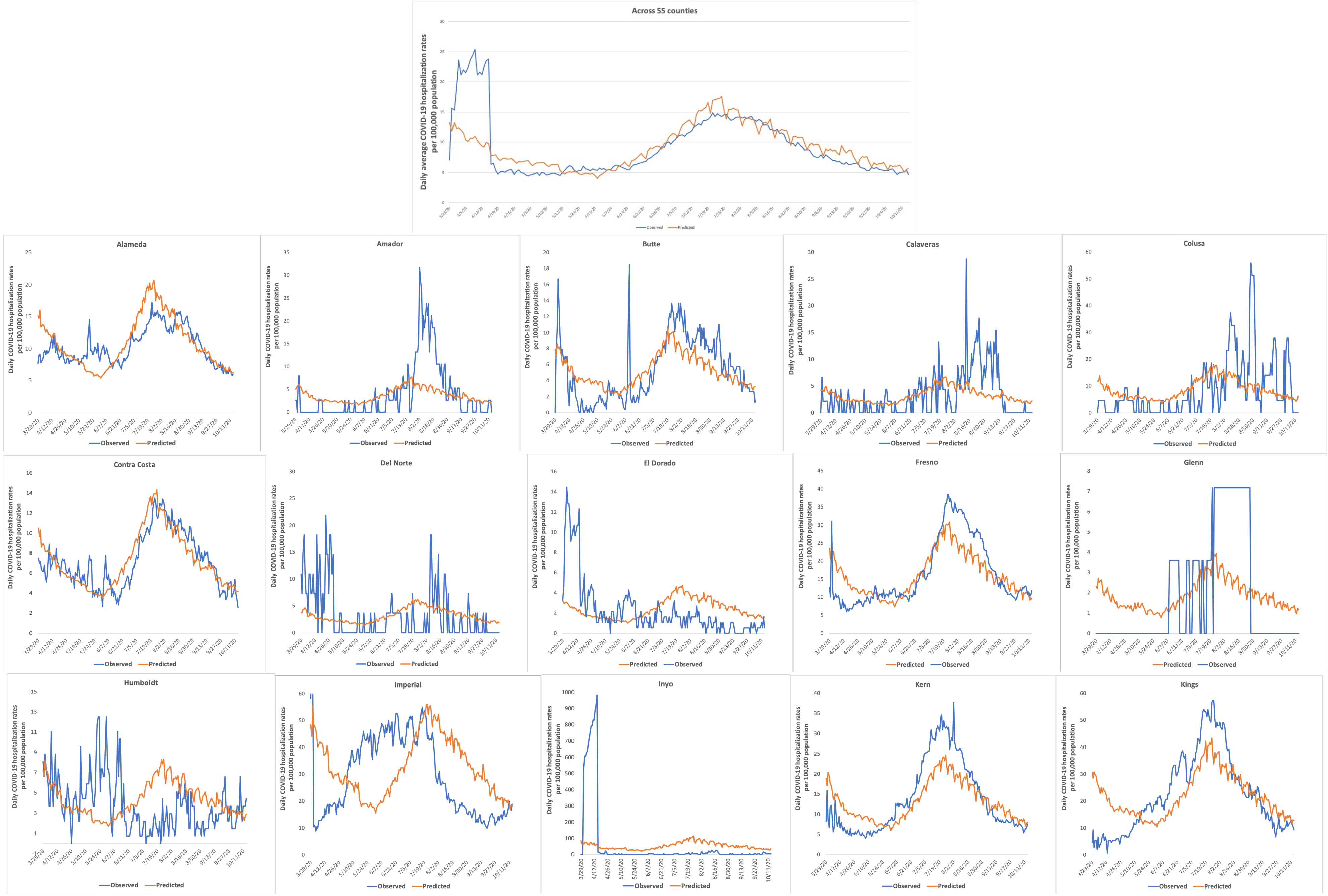

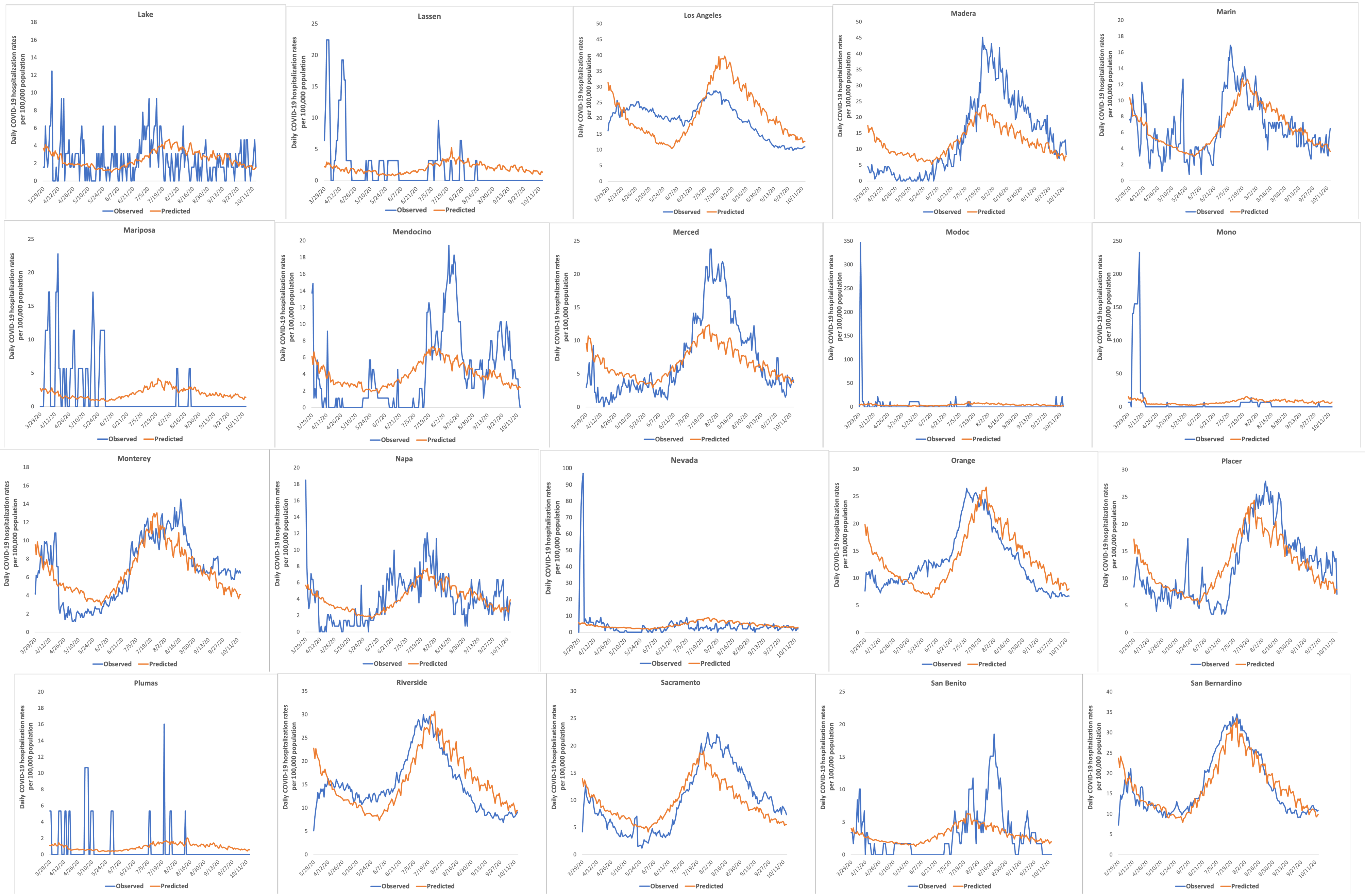
Lake
Lassen
Los Angeles
Madera
Marin
Mariposa
Mendocino
Merced
Modoc
Mono
Monterey
Napa
Nevada
Orange
Placer
Plumas
Riverside
Sacramento
San Benito
San Bernardino
Daily COVID-19 hospitalization rates per 100,000 population
3/29/20 4/12/20 4/26/20 5/10/20 5/24/20 6/7/20 6/21/20 7/5/20 7/19/20 8/2/20 8/16/20 8/30/20 9/13/20 9/27/20 10/11/20
Observed
Predicted

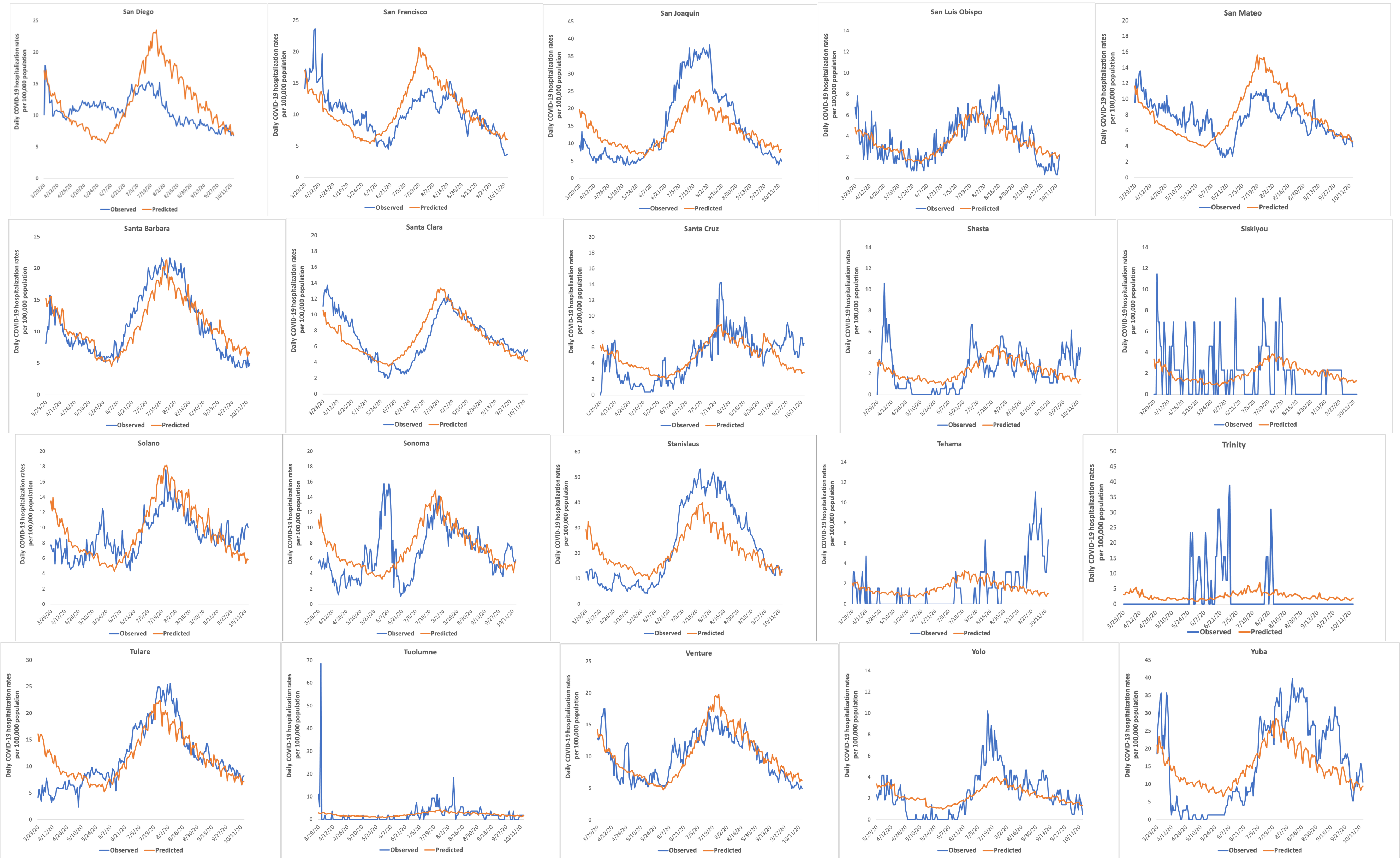
San Diego
San Francisco
San Joaquin
San Luis Obispo
San Mateo
Santa Barbara
Santa Clara
Santa Cruz
Shasta
Siskiyou
Solano
Sonoma
Stanislaus
Tehama
Trinity
Tulare
Tuolumne
Venture
Yolo
Yuba
Daily COVID-19 hospitalization rates per 100,000 population
Observed
Predicted
