# Supplementary material for: Public Protests and the Risk of Novel Coronavirus Disease Hospitalizations: A County-Level Analysis from California
Source: Int J Environ Res Public Health. 2021 Sep 8;18(18):9481. doi: 10.3390/ijerph18189481 (PMC8467497; doi:10.3390/ijerph18189481)
Supplement: Supplementary file 1 [file ijerph-18-09481-s001.zip › Supplementary Table S1.pdf]

Supplemental Table S1: Description of Count Love tag series included in author-generated protest-type categories

For racial/social justice (N=78 unique tag series)

Civil Rights; Collective Bargaining; For racial justice; For better compensation * Civil Rights; Collective Bargaining; For racial justice; For worker rights * Civil Rights; Education; For racial justice * Civil Rights; Education; For racial justice; Against police presence * Civil Rights; Education; For racial justice; For affirmative action * Civil Rights; For criminal justice reform; Prisons * Civil Rights; For greater accountability; Police * Civil Rights; For greater inclusion * Civil Rights; For racial justice * Civil Rights; For racial justice; Against business * Civil Rights; For racial justice; Against colonization * Civil Rights; For racial justice; Against colonization; Native Americans * Civil Rights; For racial justice; Against Confederate symbol * Civil Rights; For racial justice; Against white supremacy * Civil Rights; For racial justice; For continued investigation * Civil Rights; For racial justice; For criminal justice; For greater accountability; Police * Civil Rights; For racial justice; For greater accountability; Against punishment; Police * Civil Rights; For racial justice; For greater accountability; Counter protest; Police * Civil Rights; For racial justice; For greater accountability; For freedom of assembly; Police * Civil Rights; For racial justice; For greater accountability; Police * Civil Rights; For racial justice; For greater inclusion * Civil Rights; For racial justice; For transgender rights * Civil Rights; For racial justice; Martin Luther King, Jr. * Civil Rights; * For racial justice; National anthem * Civil Rights; For racial justice; Pro-LGBTQ * Civil Rights; For racial justice; Pro-LGBTQ; For greater accountability; Police * Civil Rights; For racial justice; Pro-LGBTQ; Pride * Civil Rights; For transgender rights * Civil Rights; For women's rights; For racial justice * Civil Rights; For women's rights; For racial justice; For greater accountability; Police * Civil Rights; Immigration; For racial justice; For compassionate immigration * Civil Rights; International; For racial justice; For continued investigation * Civil Rights; Pro-LGBTQ; Counter protest * Civil Rights; Pro-LGBTQ; Pride * Collective Bargaining; Against layoffs; Against punishment * Collective Bargaining; Against layoffs; Coronavirus * Collective Bargaining; Against understaffing * Collective Bargaining; For better compensation * Collective Bargaining; For better compensation; Farming * Collective Bargaining; For unionization; Amazon * Collective Bargaining; For worker rights * Collective Bargaining; For worker rights; Uber * Collective Bargaining; Healthcare; Against closure/relocation; For local services * Education; Against curriculum changes\ * Education; For firing/reassignment * Education; For tax * Education; For tax; Business * Environment; Against fossil fuels * Environment; Against hazardous conditions; Business; Air * Environment; Against hazardous conditions; Military * Environment; Against mining * Environment; Against tree removal; Utility * Executive; Against mismanagement; Post Office * Executive; Against president * Executive; Against president; Against media * Executive; Against president; Counter protest * Immigration; Against border wall * Immigration; Against ICE * Immigration; For compassionate immigration * Immigration; For compassionate immigration; Coronavirus * International; Against war * International; Against war; Armenia * International; For Palestine; Israel * Legislative; Against congressional representative * Other; Against development * Other; Against gentrification; For historic preservation * Other; Against human trafficking * Other; Against mismanagement * Other; Against mismanagement; For homeless residents * Other; Against sexual/domestic violence; For greater accountability; Military * Other; Against sexual/domestic violence; Military * Other; Against violence * Other; For affordable housing * Other; For animal welfare * Other; For homeless residents * Other; For public transportation * Other; For senior services * Other; For veteran services

Counter racial/social justice (N=14 unique tag series)

Civil Rights; For colonization * Civil Rights; For colonization; Counter protest * Civil Rights; For white supremacy * Civil Rights; For white supremacy; Counter protest
Civil Rights; Other; Against abortion rights; Against human trafficking; Conspiracy theory * Collective Bargaining; For supporting police * Executive; Against state executive * Executive; For president * Other; Against human trafficking; Conspiracy theory * Other; Against tax * Other; For conservative agenda * Other; For supporting police * Other; For supporting police; Counter protest * Other; For white supremacy; Counter protest

For pandemic intervention (N=36 unique tag series)

Collective Bargaining; Against hazardous conditions; Against understaffing * Collective Bargaining; Against hazardous conditions; Coronavirus * Collective Bargaining; Against layoffs * Collective Bargaining; Against layoffs; Against hazardous conditions; Coronavirus * Collective Bargaining; Against layoffs; Coronavirus * Collective Bargaining; For better compensation * Collective Bargaining; For better compensation; Against hazardous conditions; Coronavirus * Collective Bargaining; For better compensation; Transportation; Coronavirus * Collective Bargaining; For worker rights * Collective Bargaining; For worker rights; Against hazardous conditions; Coronavirus * Collective Bargaining; For worker rights; Coronavirus * Collective Bargaining; Healthcare; Against firing/reassignment; Against hazardous conditions; Coronavirus * Collective Bargaining; Healthcare; Against hazardous conditions * Collective Bargaining; Healthcare; Against hazardous conditions; For pandemic intervention; Coronavirus * Education; Against curriculum changes; Coronavirus * Education; Against hazardous conditions; Coronavirus * Education; Against layoffs; Coronavirus * Education; Against mismanagement; Coronavirus * Education; Against schedule change; Coronavirus * Education; For greater funding; For tax * Executive; Against budget * Executive; Against mismanagement; Coronavirus * Healthcare; Against hazardous conditions; Coronavirus * Healthcare; Against hazardous conditions; Counter protest; Coronavirus * Healthcare; For pandemic intervention; Business; Coronavirus * Healthcare; For pandemic intervention; Coronavirus * Healthcare; For pandemic intervention; Counter protest; Coronavirus * Other; Against eviction; Coronavirus * Other; Against hazardous conditions; For homeless residents; Coronavirus * Other; Against hazardous conditions; Prisons; Coronavirus * Other; Against tourism; Coronavirus * Other; For homeless residents; Coronavirus * Other; For homeless residents; Counter protest; Coronavirus * Other; For safety net; Coronavirus * Other; For safety net; Housing; Coronavirus * Other; For worker rights; For safety net; May Day; Coronavirus

Against pandemic intervention (N=12 unique tag series)

Education; Against closure/relocation; Coronavirus * Education; Against hazardous conditions; Coronavirus * Education; Against pandemic intervention; Coronavirus * Education; Against schedule change; Against pandemic intervention; Coronavirus * Executive; Against state executive; Coronavirus * Executive; Healthcare; For president; Against pandemic intervention; Coronavirus * Healthcare; Against pandemic intervention; Coronavirus * Healthcare; Against pandemic intervention; Counter protest; Coronavirus * Healthcare; For safety net; Against pandemic intervention; Coronavirus * Other; Against homeless residents; Coronavirus * Other; Against vaccines * Other; For safety net; Coronavirus

Miscellaneous (N=18 unique tag series)

Education; Against closure/relocation * Education; Against curriculum changes * Education; Against firing/reassignment * Education; For firing/reassignment * Education; For greater participation; Against hazardous conditions * International; Against foreign government; Azerbaijan * International; Against foreign government; Israel * International; Against foreign government; Philippines * International; For foreign government; Counter protest; Azerbaijan * Other; Against closure/relocation; Business * Other; Against closure/relocation; Park * Other; Against development * Other; Against punishment; Business * Other; For continued investigation; Military * Other; For criminal justice * Other; For criminal justice reform * Other; For greater accountability; Military * Other; For greater funding; For local services; Coronavirus

*Note: All protests had a series of tags (including at least 2 tags) associated with the protest, separated by ";" – we use " * " to separate the unique tag series, encompassing all tag series included in a given Protest Type category. Some tags may appear in more than one category based on associated news article for corresponding protest*
