# Supplementary material for: Public Protests and the Risk of Novel Coronavirus Disease Hospitalizations: A County-Level Analysis from California
Source: Int J Environ Res Public Health. 2021 Sep 8;18(18):9481. doi: 10.3390/ijerph18189481 (PMC8467497; doi:10.3390/ijerph18189481)
Supplement: Supplementary file 1 [file ijerph-18-09481-s001.zip › Supplementary Table S2.pdf]

Supplementary Table S2: Incidence rate ratios (IRR) and 95% Confidence Interval (95% CI) from multivariable mixed negative binomial models: Association between protests and one-week, three-week, and four-week post-protest COVID-19 hospitalization across 55 California counties, March 29th – October 14th, 2020

	**One-week post-protest COVID-19 hospitalization[1] IRR(95% CI)**		**Three-week post-protest COVID-19 hospitalization[1] IRR(95% CI)**		**Four-week post-protest COVID-19 hospitalization[1] IRR(95% CI)**	
Any protest	1.025 (0.944; 1.112)	--	0.957 (0.888; 1.032)	--	0.984 (0.914; 1.060)	--
1 protest	--	1.017 (0.941; 1.100)	--	0.959 (0.892; 1.030)	--	0.992 (0.927; 1.062)
>1 protest	--	0.951 (0.867; 1.044)	--	0.952 (0.857; 1.058)	--	0.964 (0.858; 1.082)

[1]All models adjusted for the following covariates: % of devices staying home, Healthy Places index, % with diabetes, % obese, % smokers, % male, Median age, % Hispanic, %Non-Hispanic Black or African American (Black), % Non-Hispanic American Indian/Alaska Native (AI/AN), % of urban housing units, Democratic county, and three spline variables accounting for time trend

* $p<0.05$, ** $p<0.01$, *** $p<0.001$
