# Supplementary material for: Public Protests and the Risk of Novel Coronavirus Disease Hospitalizations: A County-Level Analysis from California
Source: Int J Environ Res Public Health. 2021 Sep 8;18(18):9481. doi: 10.3390/ijerph18189481 (PMC8467497; doi:10.3390/ijerph18189481)
Supplement: Supplementary file 1 [file ijerph-18-09481-s001.zip › Supplementary Table S3.pdf]

Supplemental Table S3: Incidence rate ratios (IRR) and 95% Confidence Interval (95% CI) from multivariable mixed negative binomial models: Association between protests and two-week post-protest COVID-19 hospitalization across 51 California counties (after removing 3 counties with outliers of hospitalization), March 29th – October 14th, 2020

	All counties (N=51 counties)	
	**Model 1s**<br>**IRR(95% CI)**	**Model 2s**<br>**IRR(95% CI)**
Any protest	0.925**<br>(0.878; 0.975)	--
1 protest	--	0.926**<br>(0.879; 0.975)
>1 protest	--	0.923<br>(0.851; 1.001)
% of devices staying at home	0.987**<br>(0.977; 0.996)	0.987**<br>(0.977; 0.996)
Healthy Places Index	0.559<br>(0.177; 1.762)	0.558<br>(0.177; 1.763)
% with diabetes	1.055<br>(0.967; 1.152)	1.055<br>(0.967; 1.152)
% obese	0.956**<br>(0.924; 0.988)	0.956**<br>(0.924; 0.988)
% smokers	0.964<br>(0.799; 1.163)	0.964<br>(0.799; 1.163)
% male	0.965<br>(0.909; 1.024)	0.965<br>(0.909; 1.024)
Median age	1.030<br>(0.949; 1.119)	1.030<br>(0.949; 1.119)
% Hispanic	1.025**<br>(1.006; 1.043)	1.025**<br>(1.006; 1.043)
% Black[1]	1.077***<br>(1.035; 1.122)	1.077***<br>(1.035; 1.122)
% AI/AN[2]	0.971<br>(0.827; 1.140)	0.971<br>(0.827; 1.140)
% of urban housing units	1.018*<br>(1.002; 1.034)	1.018*<br>(1.002; 1.034)
Democratic county	0.754<br>(0.463; 1.228)	0.754<br>(0.463; 1.228)
Spline 1 (before 06/01)	0.991**<br>(0.986; 0.996)	0.991**<br>(0.986; 0.996)
Spline 2 (06/01-07/21)	1.035***<br>(1.025; 1.044)	1.035***<br>(1.025; 1.044)
Spline 3 (07/22-10/14)	0.961***<br>(0.955; 0.967)	0.961***<br>(0.955; 0.967)

[1]Non-Hispanic Black or African American; [2]Non-Hispanic American Indian/Alaska Native
* p<0.05, ** p<0.01, *** p<0.001
